# Targeted Chemotherapy Using a Cytotoxic Somatostatin Conjugate to Inhibit Tumor Growth and Metastasis in Nude Mice

**DOI:** 10.4137/cmo.s970

**Published:** 2008-08-19

**Authors:** Li-Chun Sun, L. Vienna Mackey, Jing Luo, Joseph A. Fuselier, David H. Coy

**Affiliations:** Department of Medicine, Peptide Research Laboratories, Tulane Health Sciences Center, New Orleans, LA 70112-2699, U.S.A

**Keywords:** somatostatin conjugate, somatostatin receptor, camptothecin, tumor growth, metastasis

## Abstract

The major problems of traditional chemotherapy are non-selectivity and non-specificity, resulting in severe toxic side effects. Peptides are a new-generation of drug-delivery vector to increase efficacy of this therapy and avoid the resulting damage. The cytotoxic somatostatin (SST) conjugate JF-10-81 was developed by coupling camptothecin (CPT) to the N-terminus of a SST analog (JF-07-69) using an activated carbamate linker. This conjugate selectively targets somatostatin receptor subtype 2 (SSTR2) and also retains high binding affinity and rapid internalization as well as anti-proliferative activity towards various tumor cells. JF-10-81 was tested for its inhibitory activity against the growth of human tumors which included neuroblastoma (IMR32), pancreatic cancer (CFPAC-1), leukemia (MOLT-4), pancreatic carcinoid (BON) and prostate cancer (PC-3). Both SSTR2 mRNAs and proteins were detected in all these tumor cell lines. The conjugate displayed potent *in vivo* inhibitory activity, although some of the potency measured in *in vitro* experiments was lost. JF-10-81 was found to significantly inhibit growth of these SSTR-positive tumors, resulting in 87% tumor reduction in neuroblastoma IMR32 and 97% in leukemia MOLT-4 bearing animals, even inducing regression of CFPAC-1 tumors. SSTR-overexpressing BON tumors were unfortunately relatively CPT-insensitive *in vitro*, however, JF-10-81 again exhibited *in vivo* potency presumably by specifically increasing CPT concentrations inside the tumor cells so that the inhibition rate for JF-10-81 was 85%. Also, JF-10-81 was used to treat highly invasive PC-3 tumors where s.c. injections inhibited both tumor growth (almost 60% reduction) and tumor metastasis (over 70%). This conjugate demonstrated its broad and excellent anti-tumor activity by targeting SSTR2-specific tumor tissues, supporting that short peptides and their analogs may be applied as ideal drug-delivery carriers to improve the traditional chemotherapy.

## Introduction

It is hard to overemphasize the myriad drawbacks to traditional chemotherapy used in treating advanced or metastatic cancers. However, the major problems of non-selectivity towards tumor cells and non-specificity certainly rank among the likely potential causes of severe toxic side effects. Also, the instability and/or insolubility of cytotoxic agents in aqueous media limit their clinical application. Possible routes to overcome these problems and improve their therapeutic properties are numerous and include various new drug delivery systems such as polymer [1,2], nano-particles [3–6], antibody conjugates [7,8], and short peptide hormone conjugates [9–13].

Specific receptors for many of these peptides, the most investigated being somatostatin (SST) and bombesin (BM), have been found to be expressed aberrantly in various tumors [14]. Taking advantage of these observations, receptor-targeted cytotoxic peptide conjugates can be used to target receptor-specific tumor tissues, increase efficacy of chemotherapeutic agents and decrease severe toxic side effects as long as suitable cleavable linker groups can be synthesized. Hence, several peptide conjugate candidates have been developed during recent years and short, easily synthesized peptides [13] were used as vectors to couple with chemotherapeutic agents and carry them to target receptor-specific tumor cells. In our laboratories, camptothecin (CPT), a potent anti-tumor agent [15,16], has been successfully coupled to urotensin, BM, and SST analogs. These new cytotoxic conjugates displayed relatively low toxic side effects and significant inhibition to tumor cell proliferation and tumor growth [11,12,13,17]. Also, cytotoxic conjugates of SST [18], BM [19] and luteinizing hormone-releasing hormone (LHRH) [20] coupled with 2-pyrrolinodoxorubicin, have been synthesized and investigated by others. These conjugates showed broad and excellent anti-tumor activities [18–21]. Peptide hormones as drug-delivering vectors have their own specific advantages over other approaches such as rapid internalization (via their respective GPCRs), better tumor-penetrating ability and quick circulatory clearance [14].

SSTRs, especially SSTR2, have been found to be highly expressed in various tumors [14,22,23] and tumoral blood vessels [24]. The two naturally occurring compounds, SST-14 and SST-28, bind with high affinity to all five receptor subtypes whereas certain clinically approved SST analogs only selectively bind to SSTR2 while being much more stable in the circulation. Such analogs are attractive starting points for use as vectors to precisely deliver non-selective cytotoxic agents to receptor-selective target sites. For instance, CPT coupled to a SST analog selectively bound to SSTR2 and was designed to retain high binding affinity even with the attachment of large drug molecules. This CPT-SSA conjugate JF-10-81 has already shown favorable anti-invasive and antiangiogenic activities [25]. In the current study, we further extend these studies to investigate the ability of JF-10-81 to prevent the growth of several important tumor types by sustained delivery formulations and metastasis of the highly invasive prostate cancer PC-3 tumor by periodic injections.

## Materials and Methods

### Materials

The cytotoxic CPT-SST conjugate JF-10-81 was synthesized as described previously [9]. Continuous drug-releasing pellets containing JF-10-81 (5 mg/pellet) formulated to release drug for 60 days, were made by Innovative Research of America (Sarasota, FL).

### Cell culture

The CHO-R1 and CHO-R2 cell lines, which were transfected with rat SSTR1 and SSTR2, respectively, were a kind gift from Dr. Agnes Schonbrunn (University of Texas—Houston, Houston, TX). Cells were cultured in F-12 medium containing 10% fetal calf serum. All other human tumor cell lines, including neuroblastoma IMR32, leukemia MOLT-4, pancreatic cancer CFPAC-1, prostate cancer PC-3 were purchased from ATCC (American Type Culture Collection, Manassas, VA). Human pancreatic carcinoid BON cells were a kind gift from Dr. Courtney Townsend (University of Texas—Galveston) and cultured as described previously [9,26].

### Ligand-receptor binding and internalization assay

The assay was performed as a modification of the one previously described [27,28]. The binding buffer contained Dulbecco’s Modified Eagle’s Medium (DMEM), 50 mM HEPES, 2 mM glutamine, 11.5 mM glucose, 1% bovine serum albumin (BSA) and 5 mM sodium pyruvate. The binding reactions took place in 1.5 ml eppendorf tubes containing 70–100 pM of [^125^I]Tyr^11^-SST-14 from PerkinElmer (Boston, MA 02118–2512), 1–1.5 × 10^5^ cells and the tested SST analogs (10^−5^ to 10^−13^ M) for a total volume of 300 μl. These were incubated at room temperature for 50–60 minutes and centrifuged for 1–2 min at 9,000 g. The cell pellets were washed twice with phosphate-buffered saline (PBS) and counted for total radioactivity in a Titertek gamma counter (Huntsville, AL). The analyses were done using GraphPad Prism 4.0 (San Diego, LA). For further internalization [29], cells were then re-suspended and incubated in HBSS (pH4, acetic acid) on ice for 10 min, washed with cold PBS and then re-counted for radioactivity. Due to the radioactivity of membrane-bound receptors being released by the acid wash, the remaining radioactivity represents the internalized peptide.

### RT-PCR

RT-PCR reagents were purchased from Invitrogen (Carlsbad, CA). Total RNA from the 5 different tumor cells described above was isolated. The RT-PCR amplification of the mRNAs of all 5 SSTR subtypes was performed under the same conditions as described in the reference[30] with the following primers: SSTR1-F: 5′-AAATGCGTCCCAGAAC-GGGACCT-3′, SSTR1-R: 5′-CAGGTTCTCAG-G T T G G A A G T C T T - 3 ′ ; S S T R 2 - F : 5′-GATGATCACCATGGCTGTG-3′, SSTR2-R: 5′-CAGGCATGATCCCTCTTC-3′; SSTR3-F: 5′-TCATCTGCCTCTGCTACCTG-3′, SSTR3-R: 5′-GAGCCCAAAGAAGGCAGGCT-3′; SSTR4-F: 5′-CATGGTCGCTATC-CAGTGCA-3′, SSTR4-R: 5′-GTGAGACAGAAGACGCTGGTGAACAT-3′; SSTR5-F: 5′-GCTCTTGGTGTTCGCGGACGT-3′, SSTR5-R: 5′-CAGGTTGACGATGTTGACGGT-GAAG-3′ [30]. The predicted RT-PCR product sizes are 993 bp to SSTR1, 892 bp to SSTR2, 221 bp to SSTR3, 276 bp to SSTR4 and 298 bp to SSTR5, respectively [30]. Experiments were done in triplicate.

### Western blot

The protocol was employed as described (Santa Cruz, CA). Briefly, cells were harvested, resuspended in RIPA buffer with cocktail inhibitors, homogenized by passing through a 21 gauge needle, mixed with loading buffer containing fresh DTT and heated for 5 min at 95 ^o^C. Supernatants were loaded to run on 8%–16% Tris-glycine gel after centrifugation at 10,000 g. Protein was transferred from gel to nitrocellulose membrane which was then blocked with 5% fat-free milk, washed and incubated with SSTR2 antibody (Santa Cruz). The membrane was washed again and incubated with second antibody (Santa Cruz). Eventually, films were developed according to the ECL system protocol (Amersham Biosciences, England).

### Cell proliferation assay

The kits for the cell proliferation assay (MTT Assay) were purchased from Promega (Madison, WI) and the protocol was the same as described previously [26].

### In vivo experiments

Male nude mice, 5–7 weeks of age upon arrival, were purchased from the National Cancer Institute (NCI, Frederik, MD). The animal study was approved by the Tulane University Health Sciences Center Institutional Animal Care and Use Committee (IACUC).

#### Tumor growth

The tumor tissue segments, grown from CFPAC-1, IMR32, MOLT-4 and BON cells injected into nude mice, were s.c. implanted into the flanks of nude mice as described previously [9]. All the tumors were passaged from mouse to mouse. Tumor-carrying mice then were implanted with continuous drug-releasing pellets containing 5 mg of JF-10-81.

For treatments of pancreatic cancer CFPAC-1 tumors, mice were separated into 2 groups. Each mouse in the control group was implanted with a placebo pellet. Each mouse in the test group was implanted with a pellet containing JF-10-81 (5 mg/pellet) with a drug-release time of 60 days. Tumor volumes were measured and bodyweights were taken once a week. The treatment for all other tumors (BON, IMR32 and MOLT-4) was the same as above.

#### Tumor metastasis

The experiment was performed as described [31] with modification. Human prostate cancer PC-3 cells were re-suspended in a PBS solution and further mixed with matrigel at a ratio of 1:3. The mixtures were implanted via i.p. injection at a volume of 200 ul (1 × 10^6^ cell/ml). One week later, mice were divided into 3 groups of 8–9 mice each. The control group was treated with PBS. The test groups were treated with the cytotoxic conjugate JF-10-81 (3.5 mg/kg) by i.p. or s.c. injection, 3 times a week, for a total of 12 injections. Mice were monitored and checked each week. Eventually, mice were killed by over-anaesthetization and autopsies were performed and tumor metastasis investigated.

## Results

### High binding affinity of the CPT-SST conjugate to SSTR subtype 2

**Figure 1:**
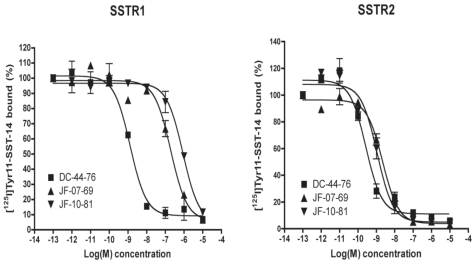
Binding affinity of native SST-14 (DC-44-76), SST analog JF-07-69 (the vector) and the conjugate JF-10-81 to stably transfected SSTR1 and 2 in CHO cells. To SSTR1, EC50 values are 1.85 (DC-44-76), 192.3 (JF-07-69) and 866.3 nM (JF-10-81), and to SSTR2, 1.45 (DC-44-76), 2.35 (JF-07-69) and 2.46 nM (JF-10-81), respectively.

Both native SST-14 (DC-44-76) acting as the control peptide and the CPT-SST conjugate JF-10-81 were tested. As expected in CHO-R1 and CHO-R2 cells, synthetic SST-14 exhibited high affinity for SSTR1 (EC50: 1.85 nM) and SSTR2 (1.45 nM) (Fig. 1), no affinity to the non-transfected CHO cells themselves. The vector JF-07-69 (2.35 nM) and its cytotoxic conjugate JF-10-81 (2.46 nM) displayed similar high affinity binding to SSTR2. However, both of them had relatively weak binding affinity to SSTR1, the EC_50_ values being 100 fold less than for SSTR2 (Fig. 1). The further internalization assay showed that the conjugate JF-10-81 could quickly internalize into the SSTR2-specific cells (data not shown). Therefore, this conjugate could potentially be applied for receptor-targeted chemotherapy.

### Expression of SSTR2 in cultured tumor cells

It has been demonstrated that SSTRs, especially SSTR2, are aberrantly expressed in many tumors [14]. Herein, the expression of mRNAs of all 5 SSTRs and protein of SSTR2 in 5 tested tumor cells were investigated by RT-PCR and western blot.

**Figure 2:**
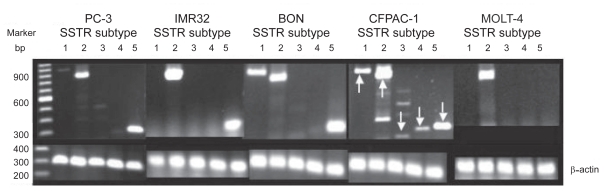
Expression of mRNAs of 5 SSTR subtypes. Total RNAs were isolated for RT-PCR amplification. The PCR products are 993 bp for SSTR1, 892 bp for SSTR2, 221 bp for SSTR3, 276 bp for SSTR4 and 298 bp for SSTR5. SSTR2 mRNA was detected in all 5 of the following human tumor cells: PC-3, IMR32, BON, CFPAC-1 and MOLT-4. The other four subtypes were undetectable in one or more tumor cell lines.

**Figure 3:**
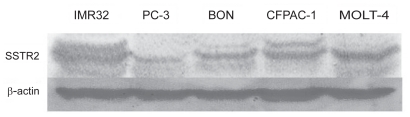
Expression of SSTR2 protein (~64 KD) detected in 5 tumor cells by western blot.

As shown in Figure 2, the results of RT-PCR demonstrated that SSTR2 and SSTR5 mRNAs existed abundantly in all 5 tumor cell lines, except for a lack of SSTR5 in MOLT-4 cells. SSTR1 was highly expressed in BON and CFPAC-1 cells. CFPAC-1 cells showed the existence of mRNAs of all 5 SSTR subtypes, with traces of one or more subtypes detectable in the other cells (Fig. 2). Further, SSTR2 proteins (~64 kD) were detected in all 5 tumor cells by western blot (Fig. 3). The expression of SSTR2 in all tested tumor cells was identical to previous reports that SSTR2 is abberantly expressed in these tumor tissues, thus confirming that SSTR2 could be used as a specific target for JF-10-81 to treat these receptor-positive tumors.

Although not easily detected in many *in vitro* cultured tumor cells, binding affinities in 5 tumor cells were further investigated with SST-14 which has high affinity to all 5 SSTR subtypes. We found that BON and IMR32 cells have higher binding affinity, with other 3 tumor cells showing weak binding to SST-14. The EC50 values were 1.45 nM to BON cells, 28.38 nM to IMR32, 195.36 nM to MOLT-4, 782.35 nM to CFPAC-1 and over 1000 nM to PC-3, respectively.

### Inhibition of the CPT-SSA conjugate to in vitro tumor cell proliferation

**Figure 4:**
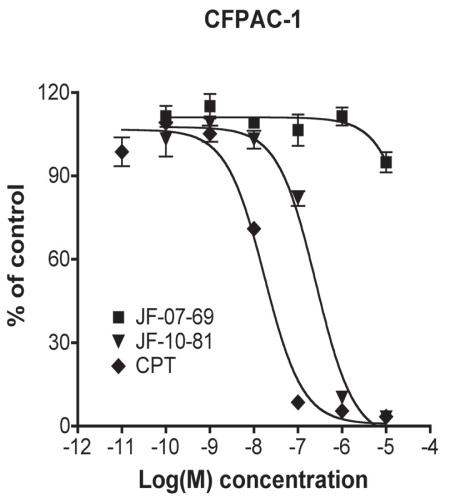
Cell proliferation assay using the CPT-SSA conjugate JF-10-81, vector JF-07-69 and CPT with human pancreatic cancer CFPAC-1 cells.

The CPT-SSA conjugate (JF-10-81) has been shown to have anti-angiogenic activity via a series of *in vitro* and *in vivo* experiments [25]. In the present study, it was tested for its inhibitory activity against five chosen SSTR-abundant human tumor cell lines, including CFPAC-1, IMR32, MOLT-4, BON and PC-3 by using the MTT assay. The EC_50_ values found were: 2.8 nM (CPT) and 46.1 nM (JF-10-81) in IMR32 cells, 2.0 nM (CPT) and 27.6 nM (JF-10-81) in MOLT-4 cells, 25.5 nM (CPT) and 196.8 nM (JF-10-81) in CFPAC-1 cells, 170.8 nM (CPT) and 1233.4 nM (JF-10-81) in BON cells, 20.8 nM (CPT) and 453.6 nM (JF-10-81) in PC-3 cells (Fig. 4 and Table 1). All the tumor cells were relatively CPT-sensitive compared to CPT-insensitive BON cells. Also, JF-10-81 exhibited the same trends as CPT itself. However, generally the conjugate partly lost its cytotoxic activity, with its EC50 being almost 10–20 fold less when compared to CPT itself. As expected, the vector peptide JF-07-69 did not work even at high concentrations (Fig. 4 and Table 1).

### Inhibition of the CPT-SSA conjugate to tumor growth and metastasis

JF-10-81 was further evaluated for its ability to inhibit tumor growth and metastasis in these SSTR2-positive tumor models mentioned above. PC-3 tumors were treated with single injections of the conjugate. The others were implanted with continuous drug-releasing pellets (5 mg/pellet). As shown in Table 2 and Figures 5 and 6, JF-10-81 (5 mg/pellet) displayed dramatic inhibitory activity in all tested tumors.

**Figure 5:**
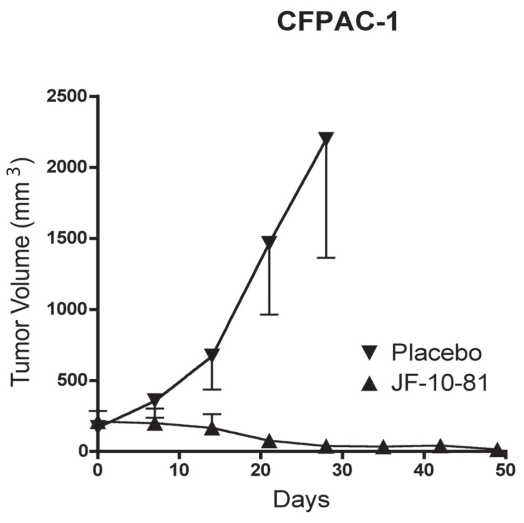
Human pancreatic cancer CFPAC-1 tumors treated with continuous drug-releasing pellets (5 mg/each) containing the conjugate JF-10-81.

In the experiment using the pancreatic cancer (CFPAC-1) tumor cells and a treatment of 5 mg/pellet/mouse, tumor volume in the control group increased from 168 ± 52 mm^3^ to 2196 ± 832 mm^3^ (up 1207%) after 4 weeks. Tumor volume in JF-10-81-treated group (5 mg/pellet) significantly decreased from 197 ± 68 mm^3^ to 44 ± 14 mm^3^ (down 78%) after 4 weeks, the inhibition rate is −92%. Tumor volumes decreased further to 8 ± 8 mm^3^ (down 96%) 7 weeks later (Fig. 5 and Table 2), with tumor disappearance in 4 out of 9 mice. Tumors were regressed with JF-10-81 treatment.

Inhibitory activity was also demonstrated in JF-10-81-treated neuroblastoma (IMR32) tumors. After 6 weeks, tumor volume sharply increased from 758 ± 292 mm^3^ to 9098 ± 4929 mm^3^ (up 1100%) in the control group, but only from 763 ± 218 mm^3^ to 1854 ± 816 mm^3^ (up 143%) in the JF-10-81-treated group (5 mg/pellet). The inhibitory rate was 87% (Table 2).

In the treatment of leukemia (MOLT-4) tumors, after 5 weeks tumor volume went from 393 ± 142 mm^3^ to 5615 ± 3143 mm^3^ (up 1328%) in the control group, but just 270 ± 89 mm^3^ to 430 ± 330 mm^3^ (up 59%) in the conjugate-treated group (5 mg/pellet). The inhibitory rate was 97% (Table 2).

Notably, in the SSTR-over-expressing, but CPT-resistant pancreatic BON tumors, JF-10-81 (5 mg/pellet) appeared to display a much more potent inhibitory activity than did CPT alone (5 mg/pellet). The percentage increase of tumor volumes was 755% in the control group, 71% in JF-10-81-treated group (5 mg/pellet) (Table 2) and 278% in the CPT-treated group (5 mg/pellet) (data not shown). The inhibitory rates for JF-10-81 and CPT treatment are 85% and 42%, respectively.

On average, JF-10-81 clearly displayed potent anti-tumor activity (depending on cell type). This could perhaps be because this conjugate concentrated CPT inside tumor cells by targeting receptors on cell surfaces, although favorable changes in the metabolism and clearance of biologically active, conjugated CPT cannot be ruled out. Even at a lower dose (1 mg/pellet), JF-10-81 was efficacious against pancreatic cancer CFPAC-1 tumor growth with the inhibitory rate reaching 56% . However, JF-10-81 at the same dose (1mg/pellet) did not work well in IMR32, MOLT-4 and BON tumor treatments (data not shown). These results also indicate that the inhibitory ability of JF-10-81 was dose-dependent.

**Figure 6:**
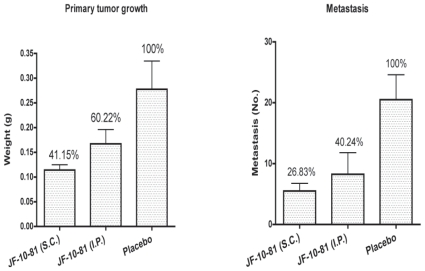
Inhibition of JF-10-81 to the highly invasive prostate cancer PC-3 tumor growth and metastasis.

The highly invasive PC-3 cells were chosen for anti-tumor metastasis experiments. PC-3 tumors in the control groups were found to metastasize to the gut, liver, chest, skin etc. of nude mice. However, in treated groups, we found that there was an obvious decrease in overall tumor metastasis, with no metastasis taking place in some mice. JF-10-81 at 3.5 mg/kg blocked almost 73% of tumor metastasis using s.c. injections and 60% by i.p. injections (Fig. 6). Meanwhile, JF-10-81 was observed to result in an almost 60% reduction of primary tumor weights using s.c. administration and 40% by i.p. injections.

## Discussion

Peptide-based receptor-targeted chemotherapy is one of a new generation of promising approaches for increasing the specificity of cancer-cytotoxin treatments. Peptides might be optimal drug-delivery vectors due to their unique advantages which have been discussed [14]. Certain peptides such as SST, BN, LHRH or their analogs have been used to deliver cytotoxic compounds by taking advantage of the peptide receptors being aberrantly expressed and sometimes over-expressed in many human tumors and in the blood vessels around tumors [13,14,22,23,24,32]. Furthermore, the structure-activity relationships of these peptides are well investigated and allow for attachment of large drug molecules using a large variety of cleavable linker chemistries with little or no loss of receptor affinity and retention of significant anti-tumor activities [9,10,20,21]. For example, AN-238, which was made by linking 2-pyrrolino-DOX to the SST analog RC-121, exhibited potent anti-tumor activity by targeting tumor cells [33,34] or tumoral blood vessels [10]. In our previous studies, cytotoxic conjugates of SST and BN analogs have revealed some utility against tumor growth and angiogenesis [9,25,35]. The CPT-SST conjugate JF-10-81 was found to be stable and readily water-soluble [9,25,36] and is predicted to display less systemic toxicity than the naked cytotoxic agent [36]. Also, JF-10-81 inhibited growth of SSTR-expressing tumors [9,11] and blocked development of angiogenesis [25]. In addition, a number of other peptide conjugates have been shown to have more specificity to tumor cells with less toxicity to normal cells [12,13,37,38].

In the present work, our most widely studied conjugate (JF-10-81) was further tested for its potentially broad anti-tumor activity using human tumors ranging through neuroblastoma, pancreatic cancer, pancreatic carcinoid, leukemia and prostate cancer. With all these tumors, as expected, RT-PCR and western blot revealed the presence of SSTR2. The internalization assay demonstrated that this SSTR2-specific JF-10-81 could quickly be taken up in SSTR2-transfected cells. In our experience, a frequent low dose was found to work better than a less frequent high dose. In a previous study, JF-10-81 did not affect human prostate cancer PC-3 tumor growth using a less frequent low dose protocol (0.73 mg/kg, twice a week) [9]. However, current results indicate that JF-10-81 obviously inhibited PC-3 tumor growth at the more frequent higher dose (3.5 mg/kg, 3 times a week), resulting in an almost 60% decrease in volume (Fig. 5). Taking these observations into account and the expectation that the targeted conjugate would have an improved therapeutic/toxicity ratio, an approach using a continuous drug-releasing pellet of 60 days was implemented. The results show that this new continuous drug-releasing approach results in significantly improved tumor responses and with no evidence of animal body weight losses. JF-10-81 resulted in an over 85% reduction in tumors of neuroblastoma IMR32 and leukemia MOLT-4 with solid tumor regression and even disappearance of some CFPAC-1 tumors occurring. As for SSTR-overexpressing, but CPT-insensitive BON tumors, JF-10-81 exhibited much more potent inhibitory effects with over double the inhibitory rate seen with the CPT formulation perhaps by increasing the CPT concentration within the vascularized solid tumor or possibly inside the tumor cells themselves (data not shown). And previously JF-10-81 also displayed anti-angiogenic activity *in vitro* [25], and now additionally showed its ability to inhibit the metastasis of the highly invasive PC-3 tumors (Fig. 5). Therefore, this conjugate JF-10-81 with optimal drug-releasing approach could be a new potential chemotherapeutics.

Reportedly, the cultured tumor cells IMR32 and CFPAC-1 highly express some SSTRs [33,39,40], with BON cells natively expressing high levels of SSTR1, SSTR2 and SSTR5 [41,42]. Our results are similar to the previous reports. Abundant SSTR2 mRNAs were detected in all 5 cultured tumor cells and SSTR2 proteins were detected as well. However, except BON and IMR32 cells, we observed little or very weak binding affinities of total 5 SSTRs in CFPAC-1, MOLT-4, PC-3 and many other tumor cells. Somatostatin binding has been demonstrated in tumor tissues taken from various primary tumors including neuroblastoma, pancreatic cancer, carcinoid and leukemia and prostate cancer [14]. Part of this problem may lie in the over passaging of commercially available cell lines and we have observed that native tumor cells and even receptor-transfected cells with initially high binding affinity can gradually lose their receptor density and binding specificity and rise non-specific binding after multiple passages. There is the possibility that cultured cells lose their natural *in vivo* micro-environments that can stimulate expression of SSTRs and their functional appearance on cell surfaces. This and additional interactions with other cells or growth factors could be part of several possible explanations for why JF-10-81 had greater *in vivo* but less *in vitro* effects than naked CPT. Additionally, in animals the conjugate would have the ability to target and kill SSTR-positive tumor blood vessels thus providing an additional mechanism for inhibition of tumor growth [10].

In conclusion, this CPT-SST conjugate has been shown to effectively inhibit angiogenesis and the progression of various SSTR-positive tumors, with low toxicity. Apparently, through targeting receptor-specific tumor sites, this kind of novel cytotoxic peptide conjugates can increase cancer cell selectivity and cytotoxicity and reduce toxic side effects relative to the traditional chemotherapy.

## Figures and Tables

**Table 1:** Effects of JF-10-81 on human tumor cell proliferation by MTT assay.

Cell Name	Cancer type	CPT	EC50 (nM) JF-10-81	JF-07-69
IMR32	Neuroblastoma	2.82 ± 0.14 (3)^a^	46.06 ± 8.47 (5)	>10,000
CFPAC-1	Pancreatic cancer	25.53 ± 5.6 (6)	196.87 ± 37 (4)	>10,000
BON	Pancreatic carcinoid	170.86 ± 14.62 (3)	1248.7 ± 114.20(3)	>10,000
PC-3	Prostate cancer	20.76 ± 2.82 (5)	453.55 ± 52.36 (3)	>10,000
MOLT-4	Leukemia	1.96 ± 0.32 (5)	27.63 ± 0.88 (6)	>10,000

a **Note:** Mean ± SEM (n) and the experiments were separately repeated n times.

**Table 2:** Effects of JF-10-81 on growth of different kinds of human tumors by treatment with continuous drug-releasing pellets.

Tumor type	Group	Dose (mg/ea)	Time (day)	Group (no./ea)	Tumor volume (mm^3^)		Inhibition (%)^c^
					Initial	Final	
Pancreatic	placebo	0	28/60^a^	10	168.12 ± 52.30^b^	2195.62 ± 832.62^b^	
CFPAC-1 tumo	JF-10-81	5	28/60 49/60	10	196.90 ± 67.96	43.58 ± 14.13 8.30 ± 7.89	−92.44%
Neuoroblastoma	placebo	0	42/60	6	758.12 ± 291.9	9097.9 ± 4928.52	
IMR32 tumor	JF-10-81	5	42/60	6	763.14 ± 217.75	1853.92 ± 815.96	86.92%
Leukemia	placebo	0	35/60	6	393.35 ± 141.92	5615.32 ± 3143.98	
MOLT-4 tumor	JF-10-81	5	35/60	6	270.27 ± 88.65	429.76 ± 329.76	96.94%
Carcinoid	placebo	0	42/60	8	58.49 ± 13.21	500.18 ± 160.68	
Bon tumor	JF-10-81	5	42/60	6	94.90 ± 17	161.6 ± 60.94	85%

a **Notes: 28/60** means that the pellet can continuously release drug for 60 days, actually, the experiments were terminated at 28 days.

b Mean ± SEM.

c Inhibition (%) = 100% *Treated Group (Final volume-Initial volume)/Control Group (Final volume-Initial volume).
